# Cell-cell junctions: a target of acoustic overstimulation in the sensory epithelium of the cochlea

**DOI:** 10.1186/1471-2202-13-71

**Published:** 2012-06-19

**Authors:** Guiliang Zheng, Bo Hua Hu

**Affiliations:** 1 Center for Hearing and Deafness, State University of New York at Buffalo, Buffalo, USA; 2 Present affiliation: Department of Otorhinolaryngology Head and Neck Surgery, Changhai Hospital, Second Military Medical University, Shanghai, China

**Keywords:** Acoustic overstimulation, Cell junctions, Cochlea, Basilar membrane, Hair cells, Dextrans, organ of Corti, Permeability

## Abstract

**Background:**

Exposure to intense noise causes the excessive movement of the organ of Corti, stretching the organ and compromising sensory cell functions. We recently revealed changes in the transcriptional expression of multiple adhesion-related genes during the acute phases of cochlear damage, suggesting that the disruption of cell-cell junctions is an early event in the process of cochlear pathogenesis. However, the functional state of cell junctions in the sensory epithelium is not clear. Here, we employed graded dextran-FITC, a macromolecule tracer that is impermeable to the organ of Corti under physiological conditions, to evaluate the barrier function of cell junctions in normal and noise-traumatized cochlear sensory epithelia.

**Results:**

Exposure to an impulse noise of 155 dB (peak sound pressure level) caused a site-specific disruption in the intercellular junctions within the sensory epithelium of the chinchilla cochlea. The most vulnerable sites were the junctions among the Hensen cells and between the Hensen and Deiters cells within the outer zone of the sensory epithelium. The junction clefts that formed in the reticular lamina were permeable to 40 and 500 but not 2,000 kDa dextran-FITC macromolecules. Moreover, this study showed that the interruption of junction integrity occurred in the reticular lamina and also in the basilar membrane, a site that had been considered to be resistant to acoustic injury. Finally, our study revealed a general spatial correlation between the site of sensory cell damage and the site of junction disruption. However, the two events lacked a strict one-to-one correlation, suggesting that the disruption of cell-cell junctions is a contributing, but not the sole, factor for initiating acute sensory cell death.

**Conclusions:**

Impulse noise causes the functional disruption of intercellular junctions in the sensory epithelium of the chinchilla cochlea. This disruption occurs at an early phase of cochlear damage. Understanding the role of this disruption in cochlear pathogenesis will require future study.

## Background

Cell-cell junctions are specialized regions of contact between the apposed plasma membranes of neighboring cells and are responsible for the maintenance of tissue architecture, cell communication, mechanical links between cells, and tissue homeostasis [1,2]. Based on their morphology, cell-cell junctions in the mammalian organ of Corti have been divided into tight, gap, adherens, and desmosome junctions [3-6]. The molecular components of these junctions in cochleae have been a focus of auditory research. Increasing evidence has implicated these molecules in the maintenance of cochlear homeostasis and in the disruption of tissue integrity in various pathological conditions [4,7-14].

In humans, noise is a common cause of acquired sensorineural hearing loss in the adult population. Exposure to intense noises, such as impulse noise, initiates cochlear damage through mechanical stresses to cellular structures [15,16]. Molecular analyses have provided evidence linking an alteration in cell adhesion to cochlear pathogenesis. In the organ of Corti, adhesion molecules participate in scar formation, a healing process involved in the maintenance of the structural integrity of the reticular lamina [4]. A recent study in our lab revealed acute changes in transcription for multiple adhesion-related genes two hours following acoustic trauma, suggesting the involvement of adhesion molecules in acute cochlear pathogenesis [14]. Moreover, mice with a conditional deficiency in the expression of vezatin, a ubiquitous adherens junction protein, exhibit an increased susceptibility to acoustic trauma [17]. This finding suggests that vezatin participates in the regulation of cochlear responses to acoustic injury. Together, these observations imply a role for the molecular response of cell-cell junctions in cochlear pathogenesis following acoustic trauma.

The molecular changes that occur within cell-cell junctions are expected to alter the structural and functional integrity of this important structure. Morphological observations have shown structural defects in the cell-cell junctions following exposure to intense noise. These defects include the detachment of sensory cells from their anchorage and the splitting of the reticular lamina at cell-cell junctions [15,16,18,19]. To date, the functional status of cell-cell junctions in the noise-traumatized cochlea is not clear. Several studies have shown signs of structural leaks in the reticular lamina, the barrier structure covering the top of the organ of Corti that protects the unique ionic environment of the organ [20-24]. While these studies indicate a disruption of the reticular lamina, it is not clear whether the dysfunction of the cell-cell junctions contributes to the observed leaks. Because junction disruption can trigger intracellular signaling pathways that lead to cell death [25,26], determining the junction integrity in noise-traumatized cochleae is essential for the understanding and interpretation of the molecular changes in cell adhesion observed following acoustic trauma.

The present study was designed to investigate the barrier function of intercellular junctions in the organ of Corti following acoustic overstimulation. To achieve this goal, we employed graded dextran-FITC probes, macromolecules that cannot penetrate cell-cell junctions, to assess the magnitude and sites of intercellular junctional disruption. We documented the sites of vulnerability for disruption and the level of damage that occurred. Importantly, we found leaks in both the reticular lamina and the basilar membrane. As junction disruption might initiate intracellular signaling pathways for regulating sensory cell damage or survival, the results presented here shed new light on the acute cochlear pathogenesis resulting from acoustic overstimulation.

## Results

### Acute sensory cell damage in noise-traumatized cochleae

**Figure 1:**
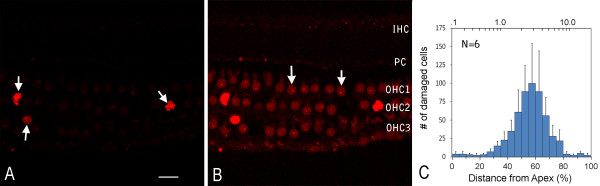
**Sensory cell damage in the organ of Corti following acoustic trauma.A,** Propidium iodide staining reveals malformed hair cell nuclei with a marked increase in fluorescence intensity (arrows). Uptake of propidium iodide into the nuclei indicates the loss of cell viability. Bar = 20 μm. **B,** Image A digitally enhanced to illustrate the weakly stained sensory cell nuclei that exhibit normal morphologies (arrows). IHC: Inner hair cells. PC: Pillar cells. OHC1, OHC2 and OHC3: The first, second, and third row of outer hair cells, respectively. **C,** The distribution of damaged sensory cells along the organ of Corti. Vertical lines above the bars represent one standard deviation. N: the number of cochleae examined.

**Figure 2:**
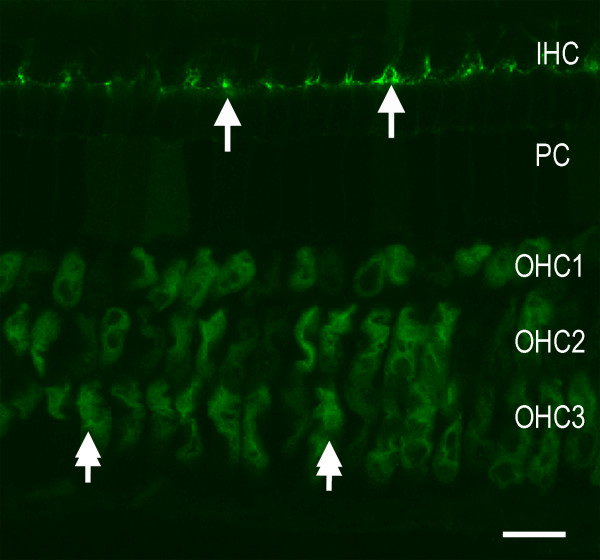
**A typical example of dextran-FITC staining in a normal organ of Corti.** All intercellular junctions among the sensory and supporting cells lack dextran-FITC fluorescence (40 kDa), except for the junctions between the inner pillar and inner hair cells, where a sporadic accumulation of dextran-FITC fluorescence is present (arrows). Outer hair cells display weak fluorescence in the cytoplasm (double-arrows). IHC: Inner hair cells. PC: Pillar cells. OHC1, OHC2 and OHC3: The first, second, and third row of outer hair cells, respectively. Bar = 25 μm.

To provide the context for interpreting the functional analysis data of cell-cell junctions, we examined the location and magnitude of sensory cell damage in the organs of Corti from six cochleae from six animals 30 minutes following noise exposure. Nuclear morphology, as illustrated by propidium iodide staining, was used as an indicator of cell damage changes in nuclear morphology are an early sign of damage and because its progression can be evaluated *via* an analysis of morphology [27,28]. We found malformed nuclei with increased propidium iodide fluorescence (Figures 1A and 2B) in the noise-damaged organs of Corti, which was distinct from the weak propidium iodide fluorescence observed in the neighboring surviving cells and in the sensory cells of normal cochleae observed in our previous studies [27,28]. Because propidium iodide is a membrane-impermeable dye, the strong uptake of dye by nuclei indicates the loss of membrane integrity in these cells, a sign of cell damage. Based on their nuclear morphology, we identified damaged sensory cells and quantified their numbers along the entire length of the organ of Corti. We found that the lesions in the hair cells were located in the sensory epithelium between the upper first and the lower second cochlear turns (Figure 1C), which in the chinchilla cochlea corresponds to a frequency range of 2–4 kHz [29]. This pattern of damage is consistent with previous observations of cochlear damage induced by similar noise conditions [30,31]. The presence of acute sensory cell damage in the organ of Corti indicates that the noise level used in the current study is able to generate acute sensory cell death.

### Dextran-FITC staining in normal organs of Corti

Lysine-fixable dextran-FITC molecules were used to assess the permeability of cell-cell junctions. These molecules bind to membrane molecules once they have leaked into junction spaces, remaining in place after fixation. Therefore, the presence of dextran-FITC fluorescence within junction regions indicates a leakage of these macromolecules into this structure.

We first examined the staining patterns of dextran-FITC in normal cochleae. Both cochleae of the animals were used, but each cochlea from each animal was treated with different molecular sizes of the dextran-FITC solutions (40, 500 or 2,000 kDa). For each size, staining was performed in four cochleae from four animals. The probe solution was surgically perfused into the perilymph space of each cochlea. For the 40 kDa dextran-FITC staining, we found no accumulation of fluorescence in the regions of intercellular junctions, except for the junctions between pillar cells and hair cells, where sporadic fluorescence was visible in certain sections of the organs of Corti (Figure 2). For the 500 and 2,000 kDa dextran-FITC staining, we found no fluorescence accumulation in any of the cell junctions (data not shown).

In normal cochleae, certain outer hair cells exhibited a weak fluorescence for dextran-FITC (40 kDa) in their cytoplasm (Figure 2). This phenomenon has been described in our previous publication [32]. The biological mechanism for the entry of dextran-FITC macromolecules is not clear, but it is likely to be related to endocytosis by the cells [33-36]. Even with this intracellular accumulation of dextran-FITC, these cells maintained their normal function as evidenced by the maintenance of auditory brainstem response thresholds of these subjects [32].

### The accumulation of dextran-FITC in cell-cell junctions of the organ of Corti following acoustic trauma

The functional status of the intercellular junctions in noise-traumatized organs of Corti was first examined using 40 kDa dextran-FITC in six cochleae from six animals. The primary site of dextran-FITC fluorescence accumulation appeared in the first and second cochlear turns, corresponding to the cochlear section that showed early sensory cell damage. Notably, the size of the areas accumulating fluorescence differed considerably between individual subjects, including no lesions (one cochlea), several small lesions (three cochleae), and a single large lesion (two cochleae). This large individual variation is consistent with the previous findings that exposure to impulse noise causes large individual variations in the magnitude of auditory functional changes [30,37].

The pattern of fluorescence distribution in the lesions was similar across individual cochleae. In the damaged regions, the accumulation of dextran-FITC fluorescence appeared in both intracellular and extracellular spaces, including cell-junction regions. Because intracellular retention of dextran-FITC in noise-traumatized cells has been reported in our previous publication [32], we focused our attention here on describing accumulation of the fluorescent tracers in the cell-cell junctions.

**Figure 3:**
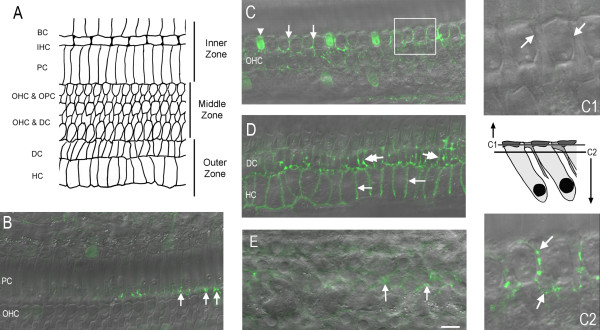
**The accumulation of dextran-FITC fluorescence (40 kDa) in noise-traumatized organs of Corti.A,** A schematic drawing showing the three structural zones in the reticular lamina: the inner zone, the middle zone, and the outer zone. Each zone has a unique set of intercellular junctions. BC: Border cells. IHC: Inner hair cells. PC: Pillar cells. OHC & OPC: Outer hair cells and outer pillar cells. OHC & DC: Outer hair cells and Deiters cells. DC: Deiters cell. HC: Hensen cells. **B,** Inner zone. To better illustrate the structure of the tissue, the image showing dextran-FITC fluorescence (green) is superimposed over the DIC view of the reticular lamina. Sporadic dextran-FITC fluorescence is present in the junctions between pillar cells and outer hair cells (arrows). **C,** Middle zone. Arrows indicate dextran-FITC fluorescence in the spaces between the hair cells and pillar cells. C1 and C2 on the right are the magnified views of the inset in *C*. The schematic plot between C1 and C2 illustrates the optical levels from which the images C1 and C2 were derived using confocal microscopy. C1 shows an absence of dextran-FITC fluorescence in the junctions between the outer hair cells and pillar cells (arrows) at the level of the reticular lamina. C2 shows the same region, but at a level that is a few microns beneath the reticular lamina, where the dextran-FITC fluorescence is present in the spaces between the outer hair cells and the pillar cells and between the outer hair cells and Deiters cells (arrows). **D,** Outer zone. The image shows the intercellular junctions between the Hensen cells (arrows) and between the Deiters cells (double-arrows). **E,** Weak fluorescence is present at the level of the Deiters cell bodies (arrows). Bar: 20 μm.

To better define the spatial distribution of the junctional disruptions observed in the six examined cochleae, we used the reticular lamina as a structural landmark to define the regions of the organ of Corti. The reticular lamina was radially divided into three zones: the inner, middle, and outer zones (Figure 3A). The inner zone contains intercellular junctions among border cells, inner hair cells, and inner and outer pillar cells. The middle zone contains the junctions between outer pillar cells and outer hair cells and between outer hair cells and Deiters cells. The outer zone contains the junctions between Deiters cells and Hensen cells and those between Hensen cells.

The three zones displayed different patterns of dextran-FITC accumulation. In the inner zone, we found no dextran-FITC fluorescence in the intercellular junctions between border and inner hair cells, except for the junctions between pillar and hair cells, where sporadic fluorescence was observed (arrows in Figure 3B). Because this fluorescence was also observed in normal cochleae, we could not attribute its presence to acoustic stress.

Strong dextran-FITC fluorescence was found in the middle zone (Figure 3C). To define the precise location of this fluorescence, we used the differential interference contrast (DIC) function of the confocal microscope to correlate the fluorescence site with the tissue structures. We found no dextran-FITC fluorescence within a depth of 3–5 μm from the top surface of the reticular lamina, which corresponds to the length of the junctions between hair and pillar cells (C1 in Figure 3). In contrast, dextran-FITC fluorescence was observed in the deeper regions, which are the Corti’s lymph-filled spaces around the bodies of outer hair and pillar cells (C2 in Figure 3). The lack of the fluorescence in the junction regions suggests that the cell junctions are not the route by which the probe enters the Corti’s lymph spaces.

In the outer zone, the cell-cell junctions between Hensen/Hensen, Deiters/Deiters, and Hensen/Deiters cells exhibited strong fluorescence (Figure 3D). The fluorescence appeared in the cell-cell contacts near the surface region (close to the surface of the reticular lamina) and also in the deeper region, suggesting the diffusion of the macromolecules within the junction spaces. Like the middle zone, the fluorescence was also distributed in the Corti’s lymph-filled spaces between the Deiters and Hensen cell bodies (data not shown). Together, the analysis of the distribution of fluorescence between the three zones of the organ of Corti suggests that the outer zone is the major site of vulnerability for cell-cell junction disruption in noise-traumatized cochleae.

In general, the fluorescence was distributed mainly in the upper portion of the organ of Corti and close to the reticular lamina. In the deep region at the level of the Deiters cell bodies, the florescence was relatively weaker (Figure 3E). The finding of strong fluorescence in the surface area of the organ of Corti suggests that the reticular lamina is the major route by which the probe enters the organ of Corti.

### The magnitude of the dextran-FITC leaks in cell-cell junctions

**Figure 4:**
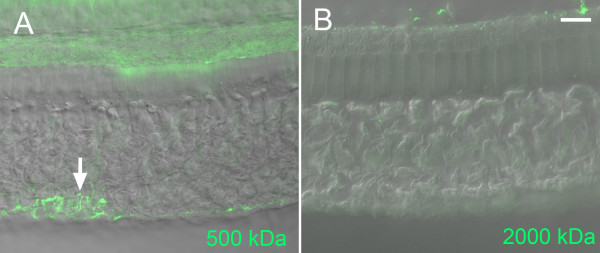
**Typical examples of the staining patterns of 500 kDa and 2,000 kDa dextran-FITC.A,** Image showing 500 kDa dextran-FITC staining. The arrow is pointing to the region of Hensen cells where strong dextran-FITC fluorescence is present. The structure that shows weak fluorescence at the top portion of the image is the tectorial membrane (indicated by the asterisk). **B,** An image showing 2,000 kDa dextran staining. No accumulation of dextran-FITC fluorescence is present in the organ of Corti. Bar: 20 μm.

Given the occurrence of cell junction leaks accommodating 40 kDa dextran-FITC molecules, we sought to determine the magnitude of the junctional dysfunction using two additional molecular sizes of molecules: 500 kDa (four normal cochleae and five noise-exposed cochleae) and 2000 kDa (four normal cochleae and three noise-exposed cochleae). We found that neither of these molecules could enter the extracellular spaces in a normal organ of Corti. Following acoustic trauma, an accumulation of 500 kDa dextran-FITC fluorescence was observed in two out of five examined cochleae (Figure 4A). Compared to the 40 kDa dextran staining, the size of the 500 kDa dextran staining in the area of the organ of Corti was smaller, and the staining appeared only in the outer zone of the junctions between Hensen cells, consistent with the finding of strong fluorescence of 40 kDa dextran-FITC in this region. When the cochleae were stained with 2,000 kDa dextran-FITC, we found no accumulation of fluorescence in the intercellular junctions of the organs of Corti (Figure 4B). Collectively, the finding that junction permeability was increased to 40 and 500 kDa, but not to 2,000 kDa, dextran-FITC molecules suggests that the cutoff size of the macromolecules that can enter junction spaces is between 40 and 2,000 kDa.

### The spatial correlation between the site of cell junction disruption and the site of acute sensory cell degeneration

**Figure 5:**
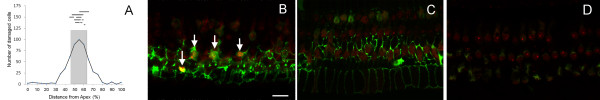
**The spatial correlation between sensory cell damage and the intercellular accumulation of Dextran-FITC.***A,* The distribution of sensory cell damage and the retention of dextran-FITC fluorescence (40 kDa) at intercellular junctions along the organ of Corti following exposure to intense noise. The line plot illustrates the distribution of sensory cell damage, and the shaded bar represents the area showing an intercellular accumulation of dextran-FITC. The horizontal bars indicate the size and the location of dextran-FITC fluorescence in the organ of Corti. Each row of the bar(s) represents the data from a single cochlea. *B,* Double-staining of dextran-FITC (green fluorescence) and propidium iodide (red fluorescence) in a section of the organ of Corti following acoustic overstimulation. The arrows point to damaged outer hair cells exhibiting malformed nuclei with increased propidium iodide fluorescence. Bar: 25 μm. *C,* A region of the organ of Corti showing an intercellular accumulation of dextran-FITC fluorescence but without propidium iodide fluorescence. *D*, A typical image of dextran-FITC and propidium iodide double-staining in a control cochlea.

Given the occurrence of acute sensory cell death following acoustic trauma, we sought to determine whether the accumulation of dextran-FITC macromolecules within the junction spaces is a consequence of cell death. To this end, we examined the spatial correlation between the site of acute cell death and the site of dextran-FITC fluorescence accumulation in cell junctions. We found that the site of intercellular junction leaks is in the upper first and lower second cochlear turns, which is where the acute sensory cell degeneration took place. However, the average size of the hair cell lesions (the section of the organ Corti exhibiting acute sensory cell death) was broader than the area showing the junction leakage (Figure 5A). Notably, the presence of dextran-FITC accumulation could be observed in both the cochlear region where dying cells were present (Figure 5B) and the region where no dying cells could be identified (Figure 5C). This observation suggests that, while both the intercellular junction disruption and acute sensory cell degeneration could occur within the same cochlear section, the two events lack a strict one-to-one spatial correlation. This suggests that cell-junction dysfunction can occur in absence of sensory cell degeneration, and conversely, sensory cell degeneration can occur in the absence of cell junction dysfunction.

### Disruption of the barrier function of the basilar membrane

The organ of Corti has two boundary structures: the reticular lamina and the basilar membrane, and both can serve as the route of dextran-FITC entry. Therefore, we proceeded to individually examine the functional integrity of these two structures. To achieve this goal, we loaded the dextran tracers (40 kDa) into either the scala vestibuli or the scala tympani. When the probe solution was perfused into the scala vestibule of noise-traumatized cochleae (n = 3 cochleae), we found a staining pattern similar to that observed for the whole-cochlea-perfused samples. Strong dextran-FITC fluorescence was present in the intercellular junctions (data not shown), suggesting that the reticular lamina is a route of dextran influx.

**Figure 6:**
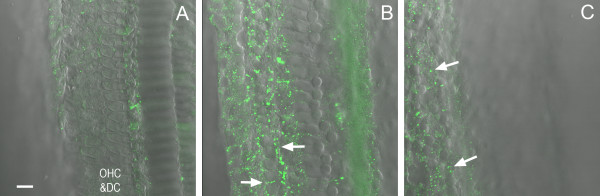
**Leaks in the basilar membrane following acoustic overstimulation.** The images were obtained from a cochlea receiving a dextran-FITC perfusion into the scala tympani. *A,* The top view of the organ of Corti at the level just beneath the reticular lamina (see the schematic drawing for the image level), where only sporadic fluorescent granules are present. Bar = 25 μm. *B,* Granular fluorescence is present in the extracellular spaces around the Deiters cells at the level beneath the outer hair cells (arrows). Asterisks mark the basilar membrane where weak dextran-FITC fluorescence is present. *C,* Image showing the staining pattern at the level of the basal portion of the Hensen cells (arrows), where sporadic fluorescence is present.

We then examined the barrier function of the basilar membrane by loading the probes into the scala tympani. In the normal cochleae (n = 2 cochleae), we found no dextran-FITC fluorescence in the organ of Corti (data not shown), indicating that the basilar membrane is impermeable to the macromolecules under physiological conditions. In the noise-traumatized cochleae (n = 4 cochleae), we found the dextran-FITC fluorescence in the extracellular spaces around Deiters cells and Hensen cells and within damaged hair cells and supporting cells (Figure 6). Compared with the upper portion of the organ of Corti (the region just beneath the reticular lamina), the deep region (the junctions among Deiters cell bases and Hensen cells) exhibited a stronger dextran-FITC fluorescence. This observation suggests that the route by which dextran-FITC enters the organ of Corti is through the basilar membrane. We also found weak fluorescence in the basilar membrane itself. However, there was no intracellular accumulation of the dextran-FITC in the mesothelial cells on the surface of the basilar membrane. Collectively, these findings indicate that the basilar membrane becomes permeable to the macromolecules after acoustic trauma.

## Discussion

The current investigation was designed to determine the functional status of intercellular junctions in the organ of Corti following acoustic trauma. We revealed a site-specific disruption of the cell-cell junctions in the outer zone of the reticular lamina of the cochlear sensory epithelium. We also documented the magnitude of the disruption and the spatial correlation between the junction disruption and sensory cell damage. Moreover, we demonstrated that the increase in permeability occurs in both the reticular lamina and the basilar membrane. This is the first study that uses macromolecular probes to investigate the functional integrity of intercellular junctions during cochlear pathogenesis following acoustic overstimulation. This study provides important information for the understanding of molecular changes in cell adhesion in response to acoustic injury.

In the current investigation, we loaded dextran-FITC probes into the perilymph space through either the scala tympani or the scala vestibuli. Compared to the application of chemical agents to the endolymph space, this approach has the merits of decreased inner ear disturbance and a greater homogenous distribution of the loaded probes within the cochlea. Moreover, it allows us to assess the barrier function of the basilar membrane. To ensure that the large molecular probes used in the current study were able to reach the endolymph space through the vestibular membrane, we examined the dextran-FITC staining in normal cochleae where the vestibular membrane is functionally intact. We found an intracellular accumulation of dextran-FITC fluorescence in sensory cells when the probes were administrated to the perilymph space. This observation is consistent with a previous finding that showed that the vestibular membrane is permeable to thorium dioxide [38], a large molecular tracer that is of comparable size to the dextran-FITC molecules used in the current study. Together, these results indicate that the vestibular membrane is permeable to macromolecular tracers, possibly through micropinocytosis [38].

The organ of Corti consists of diverse sensory and supporting cells, each forming contacts with neighboring cells. Here, we provide evidence that functional changes in intercellular junctions are site-specific following acoustic trauma. The outer zone shows a massive accumulation of tracer molecules and also displays a greater level of damage, allowing the entry of 500 kDa dextran-FITC molecules in some noise-exposed ears. This finding suggests that the outer zone is the vulnerable site for the functional disruption of cell-cell junctions in the cochlear sensory epithelium. Unlike the outer zone, accumulation of dextran-FITC fluorescence in the middle zone does not occur in the intercellular junctions, but rather in the Corti’s lymph spaces beneath the level of cell-cell junctions. This finding raises the question as to how the macromolecules reach the pericellular spaces. As demonstrated in the current study and in previous observations [15,22], acoustic trauma causes the degeneration of sensory cells. The demise of these cells can lead to structural defects in the reticular lamina, causing leaks. The dextran-FITC molecules can also diffuse from the disrupted junctions between the Hensen cells in the outer zone. Although the current study does not provide further evidence to assess the contribution of these routes of probe passage, our results suggest that the cell-cell junctions in the middle zone are less susceptible to acoustic trauma than those in the outer zone.

We estimated the magnitude of intercellular junction interruption using graded sizes of dextran-molecules and found that the cutoff size to pass through the damaged junction spaces varied between individual cochleae with the maximal leak between 40 kDa and 2,000 kDa. Based on Stoke’s radius analysis, dextrans with these molecule weights have the dimensions of approximately 14.7 to 27 nm. Because the actual sizes of the dextran macromolecules can be affected by multiple factors, including the homogeneity level of the molecules, the size of added domains (FITC), and the concentration of the working solution, the current analysis of molecular sizes can only provide a gross estimate of the magnitude of junction dysfunction.

The organ of Corti has two boundaries: the reticular lamina and the basilar membrane. While the noise-induced damage to the reticular lamina has been documented previously [18,19,22], changes in the functional integrity of the basilar membrane are largely unstudied. The basilar membrane consists of the basement membrane, filaments, homogeneous ground substance, and mesothelial cells. In the physiological condition, this structure is permeable to certain macromolecular particles [38] and has a greater permeability to small molecule agents than does the reticular lamina [39,40]. Here, we demonstrate that the basilar membrane is impermeable to dextran-FITC probes (40 kDa) and that acoustic overstimulation causes the permeabilization of the membrane. Because no intracellular accumulation of dextran-FITC fluorescence was observed within mesothelial cells, we suspect that the probes entered the organ of Corti through the mesothelial cell junctions, which are desmosome-like junctions [41]. The biological impact of leaks in the basilar membrane on sensory cell viability is not known. Considering the similarity of the ionic compositions of Corti’s lymph and the perilymph [42], we suspect that the impact of leaks in the basilar membrane is not as detrimental as the leaks in the reticular lamina that cause a mixture of endolymph and Corti’s lymph, a known factor for the induction of sensory cell degeneration [22,43].

Exposure to intense noise causes sensory cell damage in the organ of Corti. Ample evidence indicates that cell damage is initiated immediately following noise exposure and continues to develop hours, days, or even weeks afterwards [44-49]. Acute cell death features apoptotic phenotypes and can occur rapidly post-exposure [27]. Given the rapidity of acute sensory cell death, cell junction interruption, which is a direct consequence of mechanical stress, has been considered to be a triggering event for acute cell death [27]. The current finding that acute cell death is evident 30 minutes following noise exposure is consistent with the previous finding of rapid sensory cell death. We also found general agreement regarding the site of junction disruption and the site of sensory cell lesions. However, there is not a one-to-one spatial correlation between the two events. This observation suggests that the disruption of cell-cell junctions is a contributing, but not the sole, factor for the initiation of acute sensory cell death. This hypothesis is supported by a previous finding that acute apoptosis is associated with both intrinsic and extrinsic cell death pathways [50]. Future studies aimed at investigating the role of adhesion function in this multifactorial causation will shed light on the process of sensory cell degeneration induced by acoustic overstimulation.

## Conclusions

The current investigation documents the dysfunction of cell-cell junctions in the cochlear sensory epithelium following exposure to a high level of impulse noise. We demonstrate that the junction disruption is site-specific, with the most vulnerable sites at the junctions in the outer zone of the reticular lamina. We also reveal the magnitude of the junction dysfunction and identify its location at both the reticular lamina and the basilar membrane. The finding that cell adhesion disruption is an early event of cochlear damage provides insight into the understanding of molecular changes in cell adhesion induced by exposure to high-level impulse noise. This study suggests that cell junctions might serve as a therapeutic target for the prevention of noise-induced cochlear damage.

## Methods

### Animals

Adult chinchillas (450–650 g, male or female, Ryerson Chinchilla Ranch, Plymouth, OH, USA) were used in this study. They were maintained in a quiet environment with *ad lib* food and water on a 12-hour light/dark cycle. All subjects received an auditory brainstem response test for the assessment of hearing thresholds. Only the animals with normal hearing sensitivity were included in the study. The care and use of the animals reported in this study were approved by the State University of New York at Buffalo Institutional Animal Care and Use Committee.

### Noise exposure

An impulse noise was used to induce mechanical stress to cochleae. The noise signals were a series of 75 pairs of impulses (1 second between each pair), generated by a D/A converter on a signal processing board (Loughborough TMS 32020). The signals were routed through an attenuator (HP 350 D), a filter (Krohn-Hite 3550R), and a power amplifier (NAD 2200) to a loudspeaker (JBL 2360). The loudspeaker was positioned 5 cm in front of the animal’s head. The noise level was calibrated using a sound level meter (Larson and Davis 800B), a pre-amplifier (Larson and Davis model 825), and a condenser microphone (Larson and Davis, LDL 2559). The microphone was positioned at the level of the animal’s head. The peak level of the impulse noise was 155 dB peak sound pressure level (pSPL), and the total duration of the noise exposure was 75 seconds. The bandwidth of the impulse noise was 100–18,000 Hz [51].

### Macromolecular tracers for the assessment of the functional integrity of intercellular junctions

We used a macromolecular tracer, fluorescein-labeled and lysine-fixable dextrans (dextran-FITC, Invitrogen Inc.), to assess the barrier function of intercellular junctions. Because these dextran molecules have covalently bound lysine residues, they can conjugate to surrounding biomolecules by aldehyde-mediated fixation. Once leaked into the cell-cell junction, they remain in place and can be detected with fluorescence microscopy.

Three sizes of dextran-FITC (40, 500, and 2,000 kDa) were employed to assess the magnitude of the intercellular junction damage. Dextran-FITC was dissolved in an artificial perilymph solution (142 mM NaCl, 5.37 mM KCl, 1.47 mM MgCl_2_, 2 mM CaCl_2_, and 10 mM HEPES) to generate a working solution (2 mg/ml for 40 kDa dextran-FITC; 5 mg/ml for 500 kDa, and 2,000 kDa dextran-FITC).

### Dextran-FITC staining

The dextran-FITC probe was loaded into the cochlea *in vivo,* either before or after the noise exposure. When the probe solution was loaded into the cochlea before the noise exposure, the acute changes in intercellular junctions that occurred during and shortly after the noise exposure could be assessed. The surgical procedure for this experimental paradigm has been described in our previous publication [32]. Briefly, the animal was anesthetized with a mixture of ketamine (35 mg/kg) and acepromazine (0.5 mg/kg). The cochlea (either the right or the left) was accessed through a conventional posterior approach. A small opening (approximately 0.2 mm in diameter) was drilled in the bony shell over the scala tympani in the basal turn of the cochlea. Another small opening was drilled in the bony shell over the posterior semicircular canal of the same ear. Approximately 10 μl of the staining solution, containing a given molecular size of dextrans, was perfused into the cochlea through the opening in the scala tympani at the rate of 2 μl/min using a syringe pump (SP100i, World Precision Instruments). The excess perilymph and staining solution were allowed to efflux through the opening in the posterior semicircular canal. After the perfusion, the cochlear openings were closed with a tissue adhesive (Vetbond Tissue Adhesive 3 M, USA), and the bulla opening was sealed with dental cement. For the dextran-FITC staining in normal control animal, the contralateral ear underwent the same surgery for the dextran-FITC staining, but with a different size of dextran-FITC molecules. For the dextran-FITC staining in the noise-exposed animals, only one cochlea was stained for each animal. As demonstrated in our previous investigation, this surgical procedure caused only a mild shift of hearing thresholds (5–15 dB) [32]. Immediately after the loading of the probe, the animal was exposed to the impulse noise. After the noise exposure, the staining solution remained in the cochlea for 30 minutes.

The post-exposure loading of the staining solution was used in the experiment for the assessment of the permeability property of the reticular lamina and the basilar membrane separately. Because loading the probe for this set of experiments altered the cochlear mechanical property, we performed the staining procedure after the noise exposure, so that the noise impact to the cochleae could proceed.

For loading the staining solution into the scala vestibuli, the animal was anesthetized immediately after the noise exposure, and the auditory bulla was exposed using the procedure described above. Three small openings were made, one over the scala vestibuli in the basal turn of the cochlea, one over the apex at the juncture of the scala tympani and the scala vestibuli, and one over the scala tympani in the basal turn of the cochlea. Through the opening in the scala vestibuli, the dextran-FITC solution was perfused into the scala vestibuli. At the same time, the artificial perilymph solution was perfused into the scala tympani through the opening in the scala tympani. For loading the staining solution into the scala tympani, the dextran-FITC solution was perfused into the scala tympani through the opening in the scala tympani. The artificial perilymph solution was perfused into the scala vestibuli through the opening in the scala vestibuli. Concurrent perfusion of the artificial perilymph solution with the dextran-FITC solution reduced diffusion of dextran-FITC molecules from one perilymph space (either the scala vestibuli or the scala tympani) to the other. For both routes of solution applications, the perfusion lasted for 30 minutes. In addition to noise-traumatized cochleae, we examined the dextran-FITC staining in normal cochleae using a staining procedure identical to that used for the noise-exposed cochleae. The number of cochleae used for each experimental condition will be presented in the Results section.

### Assessment of sensory cell damage

To provide the context of cochlear damage for the analyses and interpretation of the results of cell junction disruption, we examined sensory cell damage in the organs of Corti at 30 min after noise exposure. Nuclear morphology was employed as the indicator of cell damage because its alteration has been recognized as an early sign of hair cell damage [27,28]. Propidium iodide was used to label cell nuclei using a method that has been described previously [48]. Briefly, after the dextran-FITC staining, the propidium iodide solution (Invitrogen Inc., 5 μg/ml) in 10 mM phosphate-buffered saline (PBS) was loaded into the cochlea and remained there for 10 min. After the staining, the cochlea was perfused with 10 mM PBS to remove the staining solution.

### Tissue collection

After the cochlear staining, the animals were sacrificed, and the cochleae were collected. Then, cochleae were fixed with 10% buffered formalin for at least 4 hours. The cochleae were dissected in 10 mM PBS, and the organs of Corti were collected. The specimens were mounted on slides containing an antifade medium (Prolong® Gold antifade reagent, Invitrogen Inc.) and examined with both fluorescence microscopy and confocal microscopy.

### Confocal imaging

The tissues were first observed with a fluorescence microscope to define the location and the size of cochlear lesions. The tissues were then examined with a confocal microscope (Zeiss LSM 510 META) capable of multiple channel imaging. This confocal imaging system enabled us to use two fluorescence channels for imaging the dextran-FITC staining and the propidium iodide staining. We also included DIC microscopy to define the tissue structures. The DIC imaging was performed simultaneously with dextran-FITC and propidium iodide imaging. With this combination of multiple channel imaging, we were able to define the tissue structures, accumulation of the dextran-FITC, and cell viability in the same area of the organ of Corti. Moreover, confocal microscopy allowed us to inspect selective cellular structures in a whole mount sample and to collect a series of sequential images for reconstruction of a three-dimensional view of the organ of Corti.

The collected images were analyzed using image processing software (Zeiss LSM Image Examiner V. 4), which allowed us not only to observe cells positioned at any specified optical section, but also to generate images showing a specified type of cells. We also used image-processing software (Image-Pro Plus 6.1) to enhance weak fluorescence. For image enhancement, the range of the gray level of the image was narrowed so that the weak fluorescence became visible (see Figure 1B). This enhancement allowed us to inspect the weakly-stained tissue structures and their orientation.

## Authors’ contributions

GZ participated in the experimental design and execution. BHH was responsible for experimental design, result interpretation, and manuscript writing. Both authors read and approved the final manuscript.
